# Characteristics and critical success factors for implementing problem-based learning in a human resource-constrained country

**DOI:** 10.4102/curationis.v38i1.1283

**Published:** 2015-08-31

**Authors:** Karen R.N. Giva, Sinegugu E. Duma

**Affiliations:** 1 Division of Nursing and Midwifery, Department of Health and Rehabilitation Sciences, University of Cape Town, South Africa.

## Abstract

**Background:**

Problem-based learning (PBL) was introduced in Malawi in 2002 in order to improve the nursing education system and respond to the acute nursing human resources shortage. However, its implementation has been very slow throughout the country.

**Objectives:**

The objectives of the study were to explore and describe the goals that were identified by the college to facilitate the implementation of PBL, the resources of the organisation that facilitated the implementation of PBL, the factors related to sources of students that facilitated the implementation of PBL, and the influence of the external system of the organisation on facilitating the implementation of PBL, and to identify critical success factors that could guide the implementation of PBL in nursing education in Malawi.

**Method:**

This is an ethnographic, exploratory and descriptive qualitative case study. Purposive sampling was employed to select the nursing college, participants and documents for review. Three data collection methods, including semi-structured interviews, participant observation and document reviews, were used to collect data. The four steps of thematic analysis were used to analyse data from all three sources.

**Results:**

Four themes and related subthemes emerged from the triangulated data sources. The first three themes and their subthemes are related to the characteristics related to successful implementation of PBL in a human resource-constrained nursing college, whilst the last theme is related to critical success factors that contribute to successful implementation of PBL in a human resource-constrained country like Malawi.

**Conclusion:**

This article shows that implementation of PBL is possible in a human resource-constrained country if there is political commitment and support.

## Introduction

Problem-based learning (PBL) is a teaching and learning strategy that has been used for more than five decades in different medical educational institutions in developed countries such as Canada, the Netherlands, the Unites States of America (USA) and other affluent countries (Archike & Nain 2005; Badeau 2010; Wood 2004). Although originally introduced into medical schools, PBL has also been adapted by other professions, including the nursing profession, and has since been extensively developed in secondary as well as primary education systems (Archike & Nain 2005; Badeau 2010; Wood 2004).

The effectiveness of PBL in problem-solving, self-directed learning, critical thinking and student motivation is widely researched and reported in medical and nursing education (Ehrenberg & Haggblom 2007; Hung, Jonassen & Liu 2006; Hwang & Kim 2006; Sangestani & Khatiban 2012). However, few African universities have implemented PBL and researched its effectiveness in medical or nursing education. These include the Faculty of Medicine at the University of Makerere in Uganda, Moi University in Kenya and the School of Nursing at the University of KwaZulu-Natal in South Africa (Gwele 1997; Iputo & Kwizera 2005; Kiguli-Malwadde *et al.* 2006).

The three examples of successful implementation of PBL in nursing and medical education in Africa mentioned above are all in fairly well resourced countries and well-established universities. They do not reflect the plight faced by human resource-constrained education institutions in countries like Malawi, where this case study was conducted.

### Problem statement

In spite of all of the measures put in place by the Government of Malawi in 2002 to ensure that PBL was implemented in nursing education, implementation of PBL in nursing education in Malawi has remained slow. By 2008 only one out of 13 nursing colleges was reported to have successfully implemented PBL. The identification of the characteristics and critical success factors required for the implementation of PBL in the identified nursing college became imperative in order to guide the implementation of PBL in other nursing colleges in Malawi.

#### Aim of the study

The aim of the study was to explore and describe the characteristics and critical success factors that facilitated implementation of PBL in one out of 13 nursing colleges as a case study in Malawi. These characteristics and critical success factors could be used to guide implementation of PBL in the whole nursing education system in Malawi and other human resource-constrained countries.

#### Background

Reports by the World Health Organization (WHO) indicate a global scarcity of health workers. The worst health worker shortages are reported in Malawi (Mangham 2007). In 2003 it was estimated that Malawi had fewer than 4000 doctors, nurses and midwives serving a population of approximately 14 million (WHO 2008). The nursing profession is the worst hit cadre of health professionals, and this is affecting the country's health sector and nursing education system. The nurses in rural areas work independently, with minimal supervision or support from doctors and senior nurse managers, since there are so few available to assist. Manafa et al.’ s (2009) study reported that nurse managers openly admitted being unable to conduct supervision because of heavy workloads. The nurses therefore need to be able to solve the health problems of the Malawian population on their own.

To address the health human resources shortages, Malawi's nursing education had to produce nurses who are able to practice independently and solve patients’ problems throughout the health sector. This could be achieved by adopting teaching and learning approaches that promote critical thinking, problem-solving skills and life-long learning amongst all new nursing graduates. PBL was therefore introduced in the nursing colleges of Malawi in 2002 to improve the nursing education system and respond to the acute nursing human resources shortage. However, in 2008 only one out of 13 nursing colleges was reported to have implemented PBL in the undergraduate nursing programme (Chalanda 2008). This highlighted the need to identify the characteristics and critical success factors necessary for implementation of PBL in the identified nursing college as a case study. This could guide the implementation of PBL in other nursing colleges where there had been delays in such implementation.

#### Research objectives

Using Owens’ (1998) sociotechnical theoretical framework, the study objectives were identified as follows: to explore and describe the goals that were identified by the college to facilitate the implementation of PBL, the resources of the organisation that facilitated the implementation of PBL, the factors related to sources of students that facilitated the implementation of PBL, and the influence of the external system of the organisation on facilitating implementation of PBL, and to identify a set of critical success factors that could guide the implementation of PBL in nursing education in Malawi.

Owens’ (1998) sociotechnical theoretical framework and its four concepts guided the objectives of the study, the research questions and data analysis. This framework views the school or the nursing college as a system with independent yet interdependent parts that mutually interact as a focused whole to achieve a goal. It recognises the interaction that exists between people and technology in workplaces, with reference to the complexity of societal structures’ interaction with human behaviour as an integrated open sociotechnical system. The sociotechnical theoretical framework has four main characteristics: goal achievement; resources for the organisation; sources of students; and the influence on the organisation. There are four concepts embedded within the four characteristics of the theory: the task subsystem, structure subsystem, technology subsystem and human subsystem (Owens 1998).

### Definition of concepts

**Characteristics** are distinguishing features or attributes of an item or a phenomenon such as implementation of PBL in the nursing college.

**Critical success factors** are factors in the nursing college under study that appeared to have determined to a greater extent the success of the implementation of the PBL programme, where it was perceived that their absence would have denied such success.

**PBL** is an educational approach that challenges students to learn to solve problems that are based on real-life situations. Emphasis is put on self-directed learning skills for students (Moore 2009).

#### Contribution to the field

The study is significant for all nursing colleges in human resource-constrained countries like Malawi, which intend to implement PBL in their programmes. They can identify the characteristics and factors that are vital to successful implementation of PBL prior to embarking on the process of implementing PBL in their nursing programmes. The use of Owens’ (1998) sociotechnical theoretical framework to understand the implementation of PBL in a nursing college adds value to nursing education research, because it highlights the important aspects that should be considered during the planning and implementation of nursing education programmes.

### Literature review

Only literature that pertains to the implementation process of PBL is addressed in this article. The success of the implementation of PBL depends on several factors, including the role played by the facilitators, competent facilitation skills, involvement of management, commitment of the staff and collaboration with stakeholders (Kiguli-Malwadde *et al.* 2006; Wells, Warelow & Jackson 2009). Other contributing factors to the success of implementation of PBL were reported as the recognition of the need to change the curriculum, the involvement of different stakeholders in the planning, collaboration with other universities, responding to government health policies, and involvement of teachers in the participatory management approach for ownership of PBL as a new philosophy and approach to learning (Kiguli-Malwadde *et al.* 2006). These studies highlight the importance of human resources for the successful implementation of PBL. Evidence shows that where there were insufficient resources, the introduction of new programmes like PBL in the curriculum are likely to fail (Sparrow & Marchington 1998:2; Wood 2004:22). However, limited resources should not prevent the resource-constrained nursing colleges from initiating changes such as the introduction of PBL in their curriculum. Rather they should consider gradual introduction of change or even look at adaptations such as a hybrid model of PBL.

The Rosario University School of Medicine in Argentina identified four basic requirements for full implementation of a PBL curriculum. These were the low number of students in each group, appropriate numbers of facilitators in specific science courses such as Sociology and clinical areas, and adequate human and financial resources (Carrera, Tellez & D’ Ottavio 2003). Facilitating small group discussions demands more human resources as facilitators and infrastructure such as classrooms. This can be a challenge in a resource-constrained country like Malawi.

In the above study it was reported that the Rosario University School of Medicine neglected to invest in the facilitators’ knowledge of PBL facilitation and other social and psychological preparation for staff and students. This resulted in resistance to change by both students and staff. Eventually the university had to adopt a hybrid PBL model to accommodate the neglected human resource aspect (Carrera *et al.* 2003). The adaptation of the original PBL or the ‘McMaster model’ to a hybrid model is permissible in order to suit the unique situations in other settings (Savin-Baden & Major 2004). This study highlighted the importance of the human subsystem in enhancing or hampering the implementation of PBL. The decision to change from implementation of full PBL to a hybrid PBL model when things were not going according to plan demonstrates to the academic leadership the importance of flexibility as a means to achieve the main goal, rather than abandoning implementation of PBL

Owens’ sociotechnical theoretical framework highlights the characteristics and sources of students as another important factor in achieving the goals of an education institution (Owens 1998). These were also reported as a critical success factor in the implementation of PBL in Makerere University where students were able to take charge of their own learning needs and to solve problems. They developed skills that enabled them to address the health problems of the community and to be successful in the job market (Kiguli-Malwadde *et al.* 2006). This could be a challenge for other resource-constrained countries where there is a poor level of general education and limited resources that promote self-directedness in learners who enter tertiary education. The students from such an educationally impoverished environment may fail to adjust to PBL.

## Research method and design

An exploratory and descriptive qualitative case study design within the ethnographic approach was used to conduct this study between May 2012 and October 2012. The design was selected because the implementation of PBL in a nursing college was a phenomenon that had not been explored and was not well understood within the nursing education system in Malawi. The ethnographic approach allowed the researcher to become part of the nursing college community in order to achieve the study objectives. The case study design provided the researcher with the real-life context of the nursing college and its boundaries (Polit & Beck 2008; Yin 2009). The identified nursing college as a single case represented a unique case out of the 13 nursing colleges which had not managed to implement PBL in Malawi.

### Context of the study

The site of the case study is one of the two campuses of the nursing college and is situated in the southern region of Malawi. The main campus is in the central region of Malawi. The nursing college offers the Bachelor's degree in Nursing and Midwifery (integrated programme), Master's degree in Midwifery, Master's degree in Community Health Nursing, Master's degree in Nursing Education, and Master's degree in Child Health in collaboration with another university in South Africa.

The capacity of the college is 300 students, about 250 of whom are undergraduates and 50 postgraduates, with about 20 lecturers. The selection of one campus and one nursing education programme, i.e. undergraduate nursing programme, helped the authors to set the boundaries and focus of the study. This is supported by Baxter and Jack (2008), who state that setting boundaries for a case study avoids an unreasonably wide scope that may bring disorder.

## Population and sampling

One nursing college was purposively selected as a unit of analysis for this case study from the population of 13 nursing colleges in Malawi. This was based on the information obtained from the minutes of the quarterly regional nursing college meetings and the periodical minutes of regional PBL workshops for nurse educators, which indicated that the identified college was the only one that had implemented PBL amongst all 13 nursing colleges in Malawi (Chalanda 2008).

The population for interviews and participant observation was all 20 academic staff of the college who were involved in both undergraduate and postgraduate teaching as well as management of the college.

The inclusion criteria for key informants were that they had to be an academic staff member of the nursing college who were in a top management position from the time of the launch of PBL implementation in 2008 to the time of conducting the study. These were likely to possess the required experience and relevant information with regard to the implementation of PBL in the nursing college. Those academic staff who were in top management positions for a period of not less than 12 months at the time of conducting the study, even if not initially there at the launch of PBL in the nursing college, were also included. It was assumed that they ought to have gained enough experience on the implementation of PBL, and thus had the relevant information needed to achieve the study objectives. Only two top management academic members were recruited as key informants.

The inclusion criteria for other participants were that they had to be undergraduate programme nurse educators who were in academic positions at the time of the launch of PBL implementation in 2002 to the time of conducting the study. Undergraduate programme nurse educators who had been involved with PBL implementation for a period of at least 12 months at the time of conducting the study were also included. It was assumed that they ought to have gained enough experience on the implementation of PBL and thus had relevant information needed by the researcher. Only seven nurse educators met the criteria and were recruited into the sample.

In total nine participants made it into the sample. These were two top management academic members and seven nurse educators. The sample size was considered adequate to provide in-depth information to answer the research question and provide insight into the study objectives. In qualitative research the number of participants is irrelevant, whilst the meaning gained from data about the phenomenon is more important (De Paulo 2000).

The inclusion criteria for documents to be reviewed were undergraduate nursing curriculum-related documents dating from at least 12 months prior to the launch of implementation of PBL to the time of conducting the study. The sampled documents comprised the PBL curriculum planning documents, curriculum documents which included course outlines, PBL modules, PBL simulations, class schedules (timetables) and assessment documents, and clinical supervision plans which included the case studies used by students.

### Pilot study

A pilot study was conducted in the first week of May 2012 to test the interview guide for appropriateness and clarity of the questions. The interview guide was revised according to constructive comments received from the pilot study participants. The questions were reduced whilst ensuring that the study objectives were retained. The pilot study data were not incorporated into the results of the main study.

### Data collection method

Data were collected by the researcher (second author) between May and October 2012, using the semi-structured interviews, participant observations, document reviews and field notes. These different sources of data complemented one another for a comprehensive understanding of the phenomenon and accomplishment of the study objectives. This triangulation of data sources guaranteed the credibility of the study findings.

The first set of data was collected from interviews with the key informants and other study participants, using the semi-structured interview guide. Field notes were taken throughout the interview sessions to capture valid points for follow up later, in order to avoid interrupting the participants’ train of thoughts whilst answering the questions (Hansen 2006).

The seven main documents that were sampled and reviewed included the undergraduate nursing curriculum; PBL simulations (documents that had sets of problem scenarios adapted from the curriculum in text form and video clips); assessment documents (e.g. portfolios, scenarios used in objective structured clinical examination); case studies used by students; classroom timetables; and minutes from a PBL staff workshop, using a checklist that was developed according to PBL facilitation requirements endorsed in literature (Archike & Nain 2005; Barrows 2000; McLoughlin 2005).

Participant observations were conducted with two nurse educators who were facilitators of PBL classes at the time for a period of six months. They were both observed during their weekly working hours, using a checklist that was developed according to PBL facilitation requirements endorsed in the literature (Archike & Nain 2005; Barrows 2000; McLoughlin 2005).

## Data analysis

Spradley’ s (1979) four steps of data analysis, including domain analysis, taxonomic analysis, componential analysis and theme analysis, were used. In the first step the data for units of cultural knowledge that are included in larger categories, depending on their similarities or semantic relationships, were searched throughout the whole data set. In the second step a system of classification of the identified domains to show the internal structure of the domain and relationships amongst the categories within the domain was decided upon. In the third step the relationships amongst the terms within a domain and the differences amongst the terms or subcategories within a domain were identified and examined. The fourth and final step involved searching and uncovering the relationships amongst domains and themes from the whole data set.

A theme map which consisted of the list of themes and subthemes was developed and used in the next step of checking the themes (Braun & Clarke 2006). Some of the themes that did not appear to generate meaningful data were discarded (Braun & Clarke 2006). The identified themes were revisited to identify similarities and differences in meaning.

Further literature from the previous studies was reviewed in order to confirm that the identified themes could be utilised as themes and enable the researcher to make the necessary inferences (Braun & Clarke 2006).

Four main themes and related subthemes were discovered according to the characteristics and the factors for successful implementation of PBL according to the nursing college as a sociotechnical system.

## Results

Nine nursing educators who taught in the undergraduate programme and were involved in the implementation of PBL participated in the study setting. Of these, two were in management positions and were regarded as the key informants. They were both females. The other seven were nursing educators responsible for teaching general nursing, midwifery and community health nursing, which are the two nursing disciplines that implemented PBL both in classroom and clinical settings in the undergraduate nursing programme. Six of these were female and one was male. This is common in the nursing profession, which is a female-dominated profession in Malawi. The teaching experience of participants ranged between five and more than ten years, with a range of two to four years of experience in PBL facilitation. Their educational backgrounds ranged from master's to doctoral degrees in different nursing specialties.

The sample of documents for the document review included the undergraduate nursing curriculum and its related documents, PBL module outlines, assessment or evaluation tools, classroom timetables, case studies and clinical supervision plans.

The four main themes were discovered from the triangulated data sources. Each theme had a number of related subthemes. The first three themes and their subthemes are related to the characteristics that were possessed by the nursing college for successful implementation of PBL. The last theme is related to the critical success factors that contribute to the successful implementation of PBL in a human resource-constrained country like Malawi.

### Theme 1: Having a goal to achieve

This theme corresponds with the main concept of Owens’ sociotechnical theoretical framework, the ‘Goal achievement’ (Owens 1998). It had the following four subthemes: developing life-long learners; review of the curriculum; embracing PBL; and gradual introduction of PBL into the curriculum.

Developing life-long learners was captured in the following extracts:

‘We wanted to develop our students to be self-directed learners for life; I think that's the thing that motivated members of staff to start PBL, we wanted to see this happening.’

‘… So we wanted to develop such life-long learners to go and work out things for themselves out there. I can say that was our main goal.’

‘We wanted the students to be as actively involved in their learning as possible; for their learning to be permanent, if I may say so, otherwise most of them would be left behind.’

The following extracts highlighted the subtheme ‘review of the curriculum’:

‘The first point for us to do this was the curriculum review for the Bachelor of Science in Nursing undergraduate programme …’

‘… curriculum review was done with the aim of incorporating the problem-based learning strategy.’

‘Stakeholders expressed the need for the college curriculum to be reviewed in response to emerging issues in the society.’

‘It is also a requirement in a democratic society that students should participate and take responsibility for their own learning, hence the review of the curriculum was necessary.’

Examples of extracts that supported the development of subtheme ‘embracing PBL’ were as follows:

‘We knew that we all had to embrace PBL as our new goal for the future.’

‘Everything has its own problems, we also had our own problems, but people were willing to take the problem-based learning with both hands and move forward with it.’

‘So we are all working towards one goal, that of embracing the PBL strategy.’

The following two extracts are examples under the subtheme ‘gradual introduction of PBL into the curriculum’:

‘We had to start small because of the challenges known to be associated with full PBL. To avoid failure, we went for the hybrid model. But also, our students are from a traditional teaching background, so we could face challenges if we went along with full PBL.’

‘We have not completely done away with the lecture method; we are not using a full PBL curriculum here. So for some content we still use lecture method since we are not yet completely established in PBL.’

### Theme 2: Resources for the organisation

This theme had two subthemes: the ‘human subsystem’ and ‘technological subsystem’; these in turn had four and two categories respectively which emerged from the data.

The subtheme ‘human subsystem’ had the following four categories: commitment of management and leadership; skills development of staff; having staff with the same basic values; and additional personnel.

Examples of extracts from data under the category ‘commitment of management and leadership’ included the following:

‘The college Management and Leadership took the leading role and actually emphasised on using PBL, encouraging everybody to change from the traditional method of teaching to this modern strategy of learning.’

‘The Dean who was in office was very much interested. Her Master's degree was in Education. She is the one who motivated most members to accept PBL.’

‘The management and leadership team was on the forefront to see that PBL is being implemented. For instance they sourced funds and material assistance from other organisations.’

The following extracts from data yielded the category ‘skills development of staff’ under the subtheme of human subsystem:

‘… the secret is educate the teacher; if not everybody can be at PhD level then people can have in-service education and that's how we have managed.’

‘Staff were trained in PBL skills, like group facilitation and planning the problem scenarios. We also had training on computer searching skills and basic computer skills.’

Examples of extracts from data under the category ‘having staff with the same basic values’ were as follows:

‘Willingness to change and a hard-working spirit have helped and teamwork has been one of the major factors that have helped in the implementation of PBL in the college.’

‘One of the values that has helped us was hard work, a hardworking spirit and teamwork.’

‘We have the culture of wanting to share, wanting to learn from each other; this has also helped us to embark on this PBL easily and we are very receptive to new things.’

The category of ‘having additional personnel’ was captured in the following examples of extracts from data:

‘There has been recruitment of more lecturers and clinical instructors recently. From last year we recruited more lecturers and clinical instructors on government secondment; this has helped a lot to beef up staff for PBL implementation.’

‘They have also made efforts to increase the human resource, for instance more IT staff were appointed to assist students and staff in the computer labs and material in order to promote the PBL implementation.’

The subtheme ‘Technological subsystem’ had three categories which included having technological equipment and material resources, staff knowledge of the curriculum, and organisation of the curriculum.

The following extracts from data are examples of the category ‘having technological equipment and material resources’ under technological subsystem as a subtheme:

‘We didn’t have a computer lab, so they renovated a room, turned it into a computer lab for students. The current computer lab you see was a classroom before.’

‘We now have a very strong library and ICT [*information and communication technology*] services; if you have to do PBL you should have resources so the students should be able to search for information … Our librarian is very resourceful to both teachers and students.’

‘Our library which has adequate books and we have computers in the computer labs and the clinical skills lab. They can also access the Internet to get information to use any time.’

The category ‘staff knowledge of the curriculum’ was captured in the following extracts from data:

‘But it was also important for staff to have knowledge of the curricular content so that they could easily assist students.’

‘Another important issue that was helpful was the fact that the facilitators had knowledge of the curriculum … yes … otherwise how do you facilitate something you don’t know?’

The following examples of extracts from data highlighted the category ‘organisation of the curriculum’:

‘In our curriculum for our students as such some of our modules have PBL scenarios but others do not have them because we are not using full PBL.’

‘The college is also using a modular system which is more on self-learning of the students.’

### Theme 3: Influence on the organisation

This theme had one subtheme, the ‘larger external system’, which had three categories: including the political influence, the social influence, and the economic influence.

The following examples of extracts from data highlighted the category ‘Political influence’:

‘In fact it was in an effort to respond to some of the priorities of the Ministry of Health, such as improving nursing education in Malawi that necessitated the implementation of PBL in our college.’

‘Most of our collaborators such as the nursing colleges in Norway, the University in Scotland, the Michigan State University, had already implemented PBL in their nursing curricula and it made our collaboration somehow uncoordinated because we needed to be speaking the same language. As a result we had to decide to implement PBL in our curriculum.’

‘The nurses’ and midwives’ council of Malawi, I mean they propose on some of the changes we want in the curriculum.’

The ‘social influence’ category came from the following examples of extracts from data:

‘The comments made by communities forced us to review our teaching methods. We identified the gaps and saw that some of the weaknesses … we decided to institute the PBL since it is known to enhance critical thinking in students. We considered such comments useful as they helped us in terms of giving us feedback on how we can improve our programmes in the college.’

‘Globally, nursing education has undergone a lot of changes including the instructional methods. Therefore, as an institution that offers nursing education we felt the social pressure not to continue with the traditional teaching methods, but to introduce PBL into our curriculum as well in order to stay abreast with our colleagues around the globe.’

The category ‘economic influence’ was highlighted through the following extracts from data:

‘Norwegian Government through the Malawi Government invested a lot of money into the initiatives for improved nursing education in Malawi project which included implementation of PBL … This initiative helped to influence this college to implement PBL.’

‘This four-day PBL workshop was organised and funded by other organisations under the Improved Health Training in Malawi – Nursing Colleges Cooperation Project.’

### Theme 4: Critical success factors

This theme had five subthemes: staff involvement in planning and communication; staff motivation and commitment of staff; collaboration with other colleges and organisations; recognition of the need for change; and provision of additional resources.

Staff involvement in planning and communication as a subtheme of critical success factors was captured from the following extracts of data:

‘… everyone was made aware of new developments. That is how it worked with the PBL for people to be involved. That has been one of the strengths where PBL implementation is concerned.’

**‘**From the word go, we started planning together for the implementation.’

**‘**The management was accommodative of our suggestions and input that we brought forward was taken with respect.’

The following extracts from data highlighted the subtheme ‘staff motivation and commitment of staff’ as a critical success factor:

‘The positive side is that people are very creative, they continue to create good resources for their students; they are very motivated and committed to PBL implementation.’

‘The college management created a supportive environment and allowed everyone to see the benefits of PBL. This was a source of inspiration and motivation for most of us.’

The following extracts from data highlighted the subtheme ‘collaboration with other colleges and organisations’:

‘Our collaboration with College of Medicine because they are ahead of us in IT [*information technology*] and e-learning has helped us, especially in training workshops on the use of IT services, and this been very crucial in our PBL implementation.’

‘Working with other institutions, like the one which is in Domasi, the education centre; they provide us with a lot of technical assistance in the PBL implementation.’

‘We relied on the Malawi Institute of Management. That helped us a lot in the implementation of PBL.’

The subtheme ‘recognition of the need for change’ was derived from the following examples from the data:

‘The staff felt that it is also time for us to change, you know … and implement PBL.’

‘Our current students are the ‘dot.com’ generation. They don’t want to be spoon-fed. They want to discover things on their own. As teachers we saw that we also have to move with the times of change and implement this PBL thing in our college.’

The subtheme ‘Provision of additional resources’ were captured from the following examples of extracts from data:

‘We got a new clinical skills lab with special equipment. We also made links with the College of Medicine where there is a multi-professional skills lab which is accessible to our students to work on their PBL activities. This additional equipment eased the PBL implementation.’

‘A new building was renovated into a computer laboratory, which has three large classrooms and a research office. That initiative really played a big role in our PBL implementation.’

‘So the planning stage was very crucial, I think we also looked at what do we want to achieve, do we have the resources in terms of the finance and human resources, that's when they decided to recruit more lecturers.’

## Ethical considerations

Ethical clearance and permission to conduct the study were obtained from the University of Cape Town Health Sciences Ethics Committee (ref: 058/2012), the National Research Health Committee of Malawi (NHSRC #1012) and the head of the institution at the case study college.

### Potential benefits and hazards

The participants were informed that there were no were no foreseeable risks associated with participation in the study. They were told that the interview or observations could be stopped at any time if they felt uncomfortable and that they were not obliged to answer questions that they did not wish to, and that their withdrawal or refusal to be observed or to answer specific questions would not have any effect on their relationship with the researcher or on their job (Hansen 2006). They were also informed that the potential benefits to the college were that the findings of the study could assist them to reflect on their successes in the implementation of PBL and identify areas for future improvements.

The researcher continuously observed the participants for signs of distress or discomfort. However, no participant distress or discomfort was observed throughout the data collection process. The participants were assured that the information obtained from them would be accessible only to the researcher and her supervisor and that there would be no chance of data being traced back to the individual participant.

### Informed consent

Informed consent was obtained after each participant had received both verbal explanations of what the study was about and the information sheet with full details of the study.

### Recruitment procedures

A list of eligible participants to the study was obtained from the gatekeepers. This list was drawn up using the inclusion and exclusion criteria. The Vice Principal and Dean of the campus under study and seven nurse educators were purposefully identified and approached for recruitment to participate in the study. They were given the information sheet and consent form. They voluntarily agreed to participate and signed the informed consent forms.

### Data protection

The names of the participants and the nursing college's name were not used on the interview transcripts or research documents. All forms of data were stored in a Microsoft computer file and backed up in an external hard drive for safety purposes. This is to ensure that they form part of the audit trail and can be retrieved at any time if required. Data will be destroyed at a later date according to the university policy.

## Trustworthiness

Prolonged engagement with participants during participant observations over a period of six months, and triangulation of data obtained from different sources, including participant interviews, observations, document reviews and field notes and member checking conducted with all participants at different levels for verification of data and confirmation of analysed data all met the credibility and dependability criteria of trustworthiness. The codes identified were checked and verified by an experienced qualitative researcher as being a true reflection of the existing codes within the data, thus meeting the confirmability criterion of trustworthiness. The audit trail of the whole research process remains available in the form of the research report, to meet the criterion of transferability.

## Discussion

The main characteristics for successful implementation of PBL in a nursing education programme in a resource-constrained country were identified in this study, and are similar to most characteristics and critical success factors that have been identified previously in affluent countries. For instance, the setting of goals that are specific for students, staff and the curriculum was reported to be important in the implementation of PBL by Barrows (2000). There should be a clearly identified goal for implementing PBL, whether the country is resource-constrained or not. It would be difficult for those involved in the implementation of PBL to support a new venture without a clearly stated goal. This is even more pertinent when resources are limited and there are other competing issues such as heavy workloads to contend with.

The awareness of one's limitations in terms of human and other resources with regard to full implementation of PBL was an important characteristic that determined the successful introduction of PBL in the case study. The nursing college opted for the gradual introduction of PBL by selecting a hybrid PBL model. This is well supported in the literature and has been used by well-established education institutions such as the University of Samford and University of KwaZulu-Natal (Gwele 1997; Major 2003). The gradual introduction of PBL as a hybrid method is also recommended by Barrows (2000) and Benson (2012), who support the use of PBL in conjunction with traditional methods of teaching where necessary.

Another important characteristic identified in the case study for successful implementation of PBL was the availability of both qualified human resources for teaching, management and administration of the programme and other resources, including good infrastructure and finances. This is supported by Savin-Baden and Murray (2000), Hoon (2003) and Yeo (2007). These authors concluded that successful implementation of PBL requires strong support from the academic administrators and the Dean, who can also seek financial support for training of staff and other resources.

Collaboration and partnership with other institutions was also found to be an important characteristic for the implementation of PBL in the case study. Similar findings have been reported elsewhere and yielded positive outcomes. For instance, Samford University collaborated with Alborg and Maastricht Universities, resulting in smooth introduction of PBL at Samford (Major 2003).

The findings revealed the teachers’ values and beliefs as important characteristics for the successful implementation of PBL in the nursing college, even when there were other competing factors like students’ poor educational background. Similar findings were reported by Hmelo-Silver and Barrows (2006), who found that if teachers’ personal goals and beliefs were not considered and adequately addressed, then implementation of PBL could be hindered. Hard work, a team spirit and willingness to embrace the PBL approach werethe main values shared by the teachers in the case study. These values are supported by Barrows (2000), who attests that the success of PBL implementation lies with a group of educators who demonstrate a willingness to work towards implementing the PBL process.

The findings further revealed that training of available staff and recruitment of others was needed for successful implementation of PBL. It was important for management to identify the level of education of existing staff members and to ensure that they were supported to obtain either Master's degrees or doctoral qualifications in different nursing specialities. This would be necessary for facilitation of PBL and designing relevant PBL scenarios. Further training in facilitation was offered to the staff who were involved with PBL facilitation. Mclean and Van Wyk (2006) support this and report that the bulk of the teaching staff in PBL programmes is formed by the facilitators. Barrows (2000) advises that management should first determine the number of staff that would be willing to engage in PBL before initiation of the PBL programme for successful implementation thereof.

Another characteristic for successful implementation of PBL identified in the case study was the availability of technological and material resources such as a well-equipped library, clinical and computer laboratories where students could access information and work on their own in the process of learning. Provision of additional technological and material resources enhanced self-directedness of the students, which increased the chances of successful implementation of PBL, even in the resource-constrained nursing college as a case study. For the acquisition of additional resources the nursing college management had to source financial assistance and support from different organisations, including the Malawi Ministry of Health and Norwegian Church Aid (2007; Chalanda 2008:2). The findings revealed that the managers also collaborated and partnered with both the Malawi Institute of Management and the College of Medicine for sharing of additional teaching and resources to supplement their limited resources. Similar findings were reported by St Joseph's Hospital in Ontario, Canada, where funding for implementation of PBL was sourced and obtained from the Ministry of Health and the Long Term Care organisation (Badeau 2010:249). Others have also identified this characteristic as a determining factor for successful implementation of PBL (Hmelo-Silver & Barrows 2008; Loyens, Magda & Rikers 2008).

These findings show that successful implementation of PBL can be achieved even in a resource-constrained country if management is creative and innovative and willing to collaborate and enter into partnerships with other education institutions for sharing of resources.

The larger external system, including the social, political and economic systems of the country, had a major influence on the successful implementation of PBL in the nursing college. The nursing college considered the feedback it received from community members regarding the graduates’ incompetency in working on their own and solving the country's population health problems; this influenced their decision to implement PBL despite human resource and financial constraints. This shows that the community has power and influence on the nursing education system. Morales-Mann and Kaitell (2001:14) support these findings and suggest that those planning the nursing education programmes should be more aware of the needs of the community. The University of Makerere in Uganda also reported that their implementation of PBL became successful because it was a response to community feedback on the quality of their graduates (Kiguli-Malwadde *et al.* 2006:129–130).

The findings reveal that implementation of PBL in the nursing college was the nursing college's response to the Government of Malawi's call to improve pre-service education in health care (Mangham 2007:2). The Government developed awareness of the country's educational needs and provided the critically needed support, as suggested by the UNESCO Chair for Problem-based Learning (2009). The findings show that another political influence to implement PBL came from the Nurses’ and Midwives’ Council of Malawi*.* The influence of the political system in the implementation of PBL in the nursing education system is emphasised by Majumda, D’ souza and Rahman (2004), who suggest that the involvement of political leaders in introduction of PBL into the educational system would help the health sector's education system to achieve a great deal.

The findings show that nursing colleges’ ability to respond positively to the external system can be a determining factor for success in the implementation of PBL, even in a resource-constrained country like Malawi.

The findings reveal that the main critical success factors for implementation of PBL in a resource-constrained country like Malawi include staff commitment, motivation and involvement in planning for the implementation of the PBL, and continuous open communication throughout the process of implementation in order to obtain their cooperation and support. This can also help to overcome the challenges of resistance to change. Chapman (2010) supports this and emphasises that when staff are aware of what is happening and are given adequate information to make them understand what is going on, they become cooperative and accept the change (Chapman 2010).

Another critical success factor for the implementation of PBL in the resource-constrained nursing college was the recognition of a need to change from old, traditional ways of teaching. This was influenced by community feedback on the graduates, awareness about the changes in global nursing and health professionals’ education, as well as the changing student profile. Despite the challenges of resource constraints, the nursing college in Malawi recognised that they are part of the global village and hence need to move with the times in order to improve the nursing practice and health outcomes of their population.

Collaboration with other organisations was identified as a critical success factor in the implementation of PBL in the resource-constrained nursing college, because it provided them with needed additional teaching and infrastructural support from well-established organisations for the benefit of their students.

The provision of additional resources was another critical success factor for the implementation of PBL in a resource-constrained country like Malawi. This called for creativity and innovation on the part of management, who went out of their way to source additional resources to augment their limited resources.

### Practical implications

This article contributes practical solutions to be considered prior to the implementation of PBL in nursing education in human resource-constrained countries. It highlights the importance of involving staff members as a critical success factor for the implementation of PBL, because this promotes ownership of the whole implementation process. It further highlights the importance of collaboration with well-resourced and well-established institutions as a critical factor for successful implementation of PBL in a human resource-constrained country.

## Limitations of the study

The limitation of the study is that it did not address the effect of the students’ background and demographic information or ‘sources of students’ as a characteristic for the successful implementation of PBL in a resource-constrained country. The ‘sources of students’ is one of the main concepts of the school or nursing college as a sociotechnical system, according to Owens’ (1998) sociotechnical framework that guided the study. The question that tried to addressthe sources of students as a factor that influences successful implementation of PBL did not yield any meaningful data.

## Recommendations

In line with the subtheme of collaboration with other colleges and organisations, we recommend that collaborative partnerships should be established with other educational institutions for sharing educational resources. Government and non-governmental organisations should also be contacted for financial support of the implementation of PBL.

In line with the subthemes on commitment of management and leadership and having staff with the same basic values, we recommend that nursing education managers and teaching staff work hand in hand from the identification of the need for implementation of PBL to its implementation, so that there is ownership of the process and cooperation from all involved. This would ensure that the teachers do not use excuses such as staff shortages as a reason for resisting change. Teacher preparation for the PBL facilitation should be included in the budget, as facilitation is a skill that should be formally acquired through training.

Future research to understand the effects of the students’ background and demographics on the successful imple­mentation of PBL in a resource-constrained country is also recommended.

## Conclusion

The implementation of PBL in a human resource-constrained country is possible if there is commitment from both management and staff to work together to achieve the goal of implementing PBL. Creativity and innovation in seeking collaborative partnerships which can provide skills development and financial support is a critical factor for successful implementation of PBL in a human resource- constrained country. Readiness for change and a positive attitude towards PBL can ensure that everybody embraces PBL implementation and does their best despite the limited resources in order to produce life-long nurses who are able to solve the health problems of their populations.

## References

[CIT0001] Archike F.I. & Nain N., 2005, ‘Promoting problem-based learning (PBL) in nursing education: A Malaysian experience’, *Nurse Education in Practice* 5, 302–311. 10.1016/j.nepr.2005.04.002 19040837

[CIT0002] Badeau C., 2010, ‘Problem-based learning: An educational method for nurses in clinical practice’, *Journal for Nurses in Staff Development* 26(6), 244–249. 10.1097/NND.0b013e31819b562c 21119376

[CIT0003] Barrows H., 2000, *Problem-based learning applied to medical education*, 2nd edn.*,* Southern Illinois School of Medicine, IL.

[CIT0004] Baxter P. & Jack S., 2008, ‘Qualitative case study methodology: Study design and implementation for novice researchers’, *Qualitative Report* 13(4), 544–559.

[CIT0005] Benson S., 2012, ‘The relative merits of PBL (problem-based learning) in university education’, *US-China Education Review* 2(4), 424–478.

[CIT0006] Braun V. & Clarke V., 2006, ‘Using thematic analysis in psychology’, *Qualitative Research in Psychology* 3(2), 77–101. 10.1191/1478088706qp063oa

[CIT0007] Carrera L.I., Tellez T.E. & D’Ottavio A.E., 2003, ‘Implementing a problem-based learning curriculum in an Argentinean medical school: Implications for developing countries’, *Academic Medicine* 78(8), 798–801. 10.1097/00001888-200308000-00010 12915370

[CIT0008] Chalanda M., 2008, *A report on the trainer of trainer*’*s workshop on problem-based learning and reflection* (unpublished), Zomba.

[CIT0009] Chapman A., 2010, *Management and leadership theories, models and gurus**,* viewed 06 August 2011, from http://www.businessballs.com/index.htm

[CIT0010] De Paulo A., 2000, *Sample size for qualitative research**,* viewed 04 October 2012, from http://www.quirks.com/articles/a2000/20001202.aspx?searchID=215035&sort=5&pg=1

[CIT0011] Ehrenberg A.C. & Haggblom M., 2007 ‘Problem-based Learning in clinical nursing education: Integrating theory into practice’, *Nurse Education in Practice* 7, 67–74. 10.1016/j.nepr.2006.04.005 17689426

[CIT0012] Gwele N.S., 1997, ‘The development of staff concerns during implementation of problem-based learning’, *Nursing Medical Teacher* 19(4), 275–284. 10.3109/01421599709034205

[CIT0013] Hansen E.C., 2006, *Successful qualitative health research: A practical introduction*, South Wind Production, Singapore.

[CIT0014] Hmelo-Silver C.E. & Barrows H.S., 2006, ‘Goals and strategies of a problem-based learning facilitator’*, Interdisciplinary Journal of Problem-based Learning* 1(1), viewed 12 November 2011, from 10.7771/1541-5015.1004

[CIT0015] Hmelo-Silver C.E. & Barrows H.S., 2008, ‘Facilitating collaborative knowledge building’, *Cognition and Instruction* 26, 48–94. 10.1080/07370000701798495

[CIT0016] Hoon K., 2003, ‘Implementation of problem-based learning in Asian medical schools and students’ perceptions of their experience’, *Medical Education* 37(5), 401–409. 10.1046/j.1365-2923.2003.01489.x 12709180

[CIT0017] Hung W., Jonassen D.H. & Liu R., 2006, *All problems are not equal: Implications for problem-based learning*, viewed 04 October 2011, from http://www.aect.org/edtech/edition3/ER5849X-C038.fm.pdf

[CIT0018] Hwang S.W & Kim M.J., 2006, ‘A comparison of problem-based learning and lecture-based learning in an adult health nursing course’, *Nurse Education Today* 26, 315–332. 10.1016/j.nedt.2005.11.002 16364510

[CIT0019] Iputo J.E. & Kwizera E., 2005, ‘Problem-based learning improves the academic performance of medical students in south Africa’, *Medical Education* 39, 388–393, viewed 04 October 2012, from onlinelibrary.wiley.com/doi/10.1111/j.1365-2929. 2005.02106 15813761 10.1111/j.1365-2929.2005.02106.x

[CIT0020] Kiguli-Malwadde E., Kijjambu S., Kiguli S., Galukande M., Mwanika A. Luboga S. et al., 2006, ‘Problem based learning, curriculum development and change process at faculty of medicine, Makerere University, Uganda’, *African Health Sciences* 6(2), 127–130. 16916306 10.5555/afhs.2006.6.2.127 PMC1831972

[CIT0021] Loyens S.M.M., Magda R. & Rikers R.M.J.P., 2008, ‘Self-directed learning in problem-based learning and its relationships with self-regulated learning’, *Educational Psychology Review* 20, 411–427. 10.1007/s10648-008-9082-7

[CIT0022] Major C.H., 2003, ‘Problem-based learning in general education at Samford University: A case study of changing faculty culture through targeted improvement efforts’, *Journal of General Education* 51(4), 235–256. 10.1353/jge.2003.0015

[CIT0023] Majumda A.A., D'souza A. & Rahman S., 2004, ‘Trends in medical education: Challenges and directions for need-based reforms of medical training in South-East Asia’, *Indian Journal of Medical Sciences* 58(9), 369–380. 15470278

[CIT0024] Manafa O., McAuliffe E., Maseko F., Bowie C., MacLachlan M. & Normand C., 2009, ‘Retention of health workers in Malawi: Perspectives of health workers and district management’, *Human Resources for Health*, viewed 03 October 2012, from http://www.human-resources-health.com/content/7/1/65 10.1186/1478-4491-7-65 PMC2722569 19638222

[CIT0025] Mangham L., 2007, *Addressing the human resource crisis in Malawi*’*s health sector: Employment preferences of public sector registered nurses* (ESAU Working Paper 18), Overseas Development Institute, London.

[CIT0026] Mclean M. & Van Wyk J., 2006, ‘Twelve tips for recruiting and retaining facilitators in a problem-based learning programme’, *Medical Teacher* 28(8): 675–679. 10.1080/01421590601110033 17594576

[CIT0027] McLoughlin J., 2005 ‘Co-ordinating and managing PBL programmes: Challenges and strategies’ in Barrett T., MacLabhrainn I. & Fallon H. (eds.), *Handbook of Enquiry and Problem-based Learning; Irish case studies and international perspectives*, AISHE & NUI Galway, Galway.

[CIT0028] Moore T.S., 2009, ‘The student tutor experience in a problem-based learning course: A case study’, PhD thesis, University of Northern Arizona.

[CIT0029] Morales-Mann E.T. & Kaitell C.A., 2001, ‘Problem-based learning in a new Canadian curriculum’, *Journal of Advanced Nursing* 33(1), 13–19. 10.1046/j.1365-2648.2001.01633.x 11155104

[CIT0030] Norwegian Church Aid, 2007, *Country Programme Plan Malawi*, viewed 11 June 2011, from www.kirkensnodhjelp.no/Documents/Kirken

[CIT0031] Owens R.G., 1998, *Organisational behaviour in education**,* 6th edn., Prentice-Hall, Needham Heights, MA.

[CIT0032] Polit D.F. & Beck C.T., 2008, *Nursing research: Generating and assessing evidence for nursing Practice*, Lippincott Williams & Wilkins, Philadelphia, PA.

[CIT0033] Sangestani G. & Khatiban M., 2012, ‘Comparison of problem-based learning and lecture-based learning in midwifery’, *Nurse Education Today* 33(8), 791–795, viewed 25 September 2012, from http://www.sciencedirect.com/science/article/pii/S0260691712000925# 22503681 10.1016/j.nedt.2012.03.010

[CIT0034] Savin-Baden M. & Major C.H., 2004, *Foundations of problem-based learning**,* Open University Press, Maidenhead.

[CIT0035] Savin-Baden M. & Murray I., 2000, ‘Staff development in problem-based learning’, *Teaching in Higher Education* 5(1), 107–126. 10.1080/135625100114993

[CIT0036] Sparrow P. & Marchington M., 1998, *Barriers to change individual and organisational barriers to change**,* viewed 07 September 2011, from http://robabdul.com/barrierstochange-individual-organisational-barriers-to-change.asp

[CIT0037] Spradley J.P., 1979, *The ethnographic interview**,* Holt Reinhart & Winston, Orlando, FL.

[CIT0038] UNESCO Chair for Problem-Based Learning, 2009, *Presentation* at a National Symposium, Aalborg, viewed 03 August 2011, from http://www.mpbl.aau.dk

[CIT0039] Wells S.H., Warelow P.J. & Jackson K.L., 2009, ‘Problem based learning: A conundrum’, *Contemporary Nurse* 33(2), 191–201. 10.5172/conu.2009.33.2.191 19929163

[CIT0040] Wood D.F., 2003, ‘ABC of learning and teaching in medicine’, *British Medical Journal* 326, 328–330. 10.1136/bmj.326.7384.328 12574050 PMC1125189

[CIT0041] Wood E.J., 2004, ‘Problem-based learning’, *Acta Biochimica Polonika* 51(2), 21–26. 15457645

[CIT0042] World Health Organization, 2008, *A country case study: Malawi*’*s emergency human resources programme**,* World Health Organization, Geneva.

[CIT0043] Yeo R., 2007, ‘Problem-based learning: A viable approach in leadership development?’ *International Journal of Educational Management* 19(7), 541–551. 10.1108/09513540510625581

[CIT0044] Yin R., 2009, *Case study research design and methods**,* 4th edn., Sage Publications, Los Angeles, CA.

